# Effect of c-Abl tyrosine kinase on the cellular response to paclitaxel-induced microtubule damage

**DOI:** 10.1054/bjoc.2000.1440

**Published:** 2000-10-26

**Authors:** A Nehmé, B L Lee, R Baskaran, Q Zhang, X Lin, R D Christen

**Affiliations:** Department of Medicine, University of California, 9500 Gilman Drive, San Diego, La Jolla, CA, 92093–0058, USA; Department of Anatomy, College of Medicine, Seoul National University, 28 Yeongon-Dong, Chongno-Gu, Seoul, 110–799, Korea; Department of Molecular Genetics and Biochemistry, University of Pittsburgh Medical Center, Pittsburgh, PA-15261, USA

**Keywords:** c-Abl tyrosine kinase, paclitaxel, p21^WAF1^, p53, cell cycle

## Abstract

DNA damage has been shown to activate c-Abl tyrosine kinase. We now report that, in addition to DNA damage, microtubule damage induced by paclitaxel results in activation of c-Abl kinase. In 3T3 cells, the presence of c-Abl kinase increased paclitaxel-induced cell death. In Abl-proficient cells, paclitaxel produced a marked and prolonged G2/M arrest which peaked at 24 h and a rapid and marked induction of p21^WAF1^which also peaked at 24 h. In Abl-deficient cells, the G2/M arrest induced by paclitaxel was less prominent and shorter in duration and the effect of paclitaxel on p21^WAF1^expression was reduced and delayed. Paclitaxel had no effect on p53 expression and MAPK phosphorylation. These findings indicate that, in 3T3 cells, c-Abl kinase facilitates cell death and regulates G2/M arrest in response to paclitaxel-induced microtubule damage in a pathway that is dependent on p21^WAF1^and independent of MAPK activity. © 2000 Cancer Research Campaign


*British Journal of Cancer* (2000) **83**(10), 1360–1366
© 2000 Cancer Research Campaign
doi: 10.1054/ bjoc.2000.1440, available online at http://www.idealibrary.com on IDEAL®

### Effect of c-Abl tyrosine kinase on the cellular response to paclitaxel-induced microtubule damage

**A Nehmé[1], BL Lee[2], R Baskaran[3], Q Zhang[1], X Lin[1] and RD Christen[1]**

[1]Department of Medicine, University of California, San Diego, 9500 Gilman Drive, La Jolla CA 92093–0058, USA; [2]Department of Anatomy, College of Medicine, Seoul National University, 28 Yeongon-Dong, Chongno-Gu, Seoul 110–799, Korea; [3]Department of Molecular Genetics and Biochemistry, University of Pittsburgh Medical Center, Pittsburgh PA-15261, USA

**Summary** DNA damage has been shown to activate c-Abl tyrosine kinase. We now report that, in addition to DNA damage, microtubule damage induced by paclitaxel results in activation of c-Abl kinase. In 3T3 cells, the presence of c-Abl kinase increased paclitaxel-induced cell death. In Abl-proficient cells, paclitaxel produced a marked and prolonged G2/M arrest which peaked at 24 h and a rapid and marked induction of $p21^{WAF1}$ which also peaked at 24 h. In Abl-deficient cells, the G2/M arrest induced by paclitaxel was less prominent and shorter in duration and the effect of paclitaxel on $p21^{WAF1}$ expression was reduced and delayed. Paclitaxel had no effect on p53 expression and MAPK phosphorylation. These findings indicate that, in 3T3 cells, c-Abl kinase facilitates cell death and regulates G2/M arrest in response to paclitaxel-induced microtubule damage in a pathway that is dependent on $p21^{WAF1}$ and independent of MAPK activity. © 2000 Cancer Research Campaign

**Keywords:** c-Abl tyrosine kinase; paclitaxel; $p21^{WAF1}$; p53; cell cycle c-Abl is a non-receptor tyrosine kinase that is related to Src-homology domains 2 and 3 (SH2 and SH3) protein kinases (Wang, 1993). The finding that c-Abl associates with the retinoblastoma (Rb) protein has supported a role for c-Abl kinase in regulating the cell cycle (Kipreos and Wang, 1992). Other studies have demonstrated that c-Abl kinase phosphorylates the C-terminal domain of RNA polymerase II (Baskaran et al, 1996) and stimulates transcription (Kipreos and Wang, 1992).

Recent work has demonstrated that DNA damage induced by a variety of agents, including ionizing radiation, cisplatin, mitomycin C, methyl methane sulfonate, and cytarabine results in activation of c-Abl kinase (Kharbanda et al, 1995*a*; 1995b; Liu et al, 1996; Nehme et al, 1997). In response to DNA damage, c-Abl associates with DNA-dependent protein kinase (DNA-PK) and is activated by DNA-PK-dependent phosphorylation. Components of the DNA-PK complex are involved in the sensing and repair of DNA double-strand breaks.

The ATM protein has been shown to serve as an important link between DNA damage and activation of c-Abl kinase (Baskaran et al, 1997; Shafman et al, 1997). When cells are treated with ionizing radiation, c-Abl is activated, but only in the presence of functional ATM. The association between ATM and c-Abl seems to be constitutive; however, ATM activates c-Abl tyrosine kinase activity only following DNA damage.

Activation of c-Abl kinase has been shown to directly contribute to the regulation of growth arrest induced by ionizing radiation (Yuan et al, 1996). c-Abl has been found to interact with p53 and Rb (Goga et al, 1995; Welch and Wang, 1993). c-Abl activates the transactivation function of p53 and thus stimulates the induction of $p21^{WAF1}$ (Goga et al, 1995). In response to DNA damage, c-Abl contributes to growth arrest by a p53-dependent and $p21^{WAF1}$-independent mechanism (Kharbanda et al, 1998).

It was our hypothesis that DNA damage and microtubule damage result in activation of similar signaling pathways. Since DNA damage activates c-Abl kinase, we asked whether c-Abl kinase is involved in the signaling cascade activated by microtubule damage. We therefore determined the effects of the microtubule-damaging agent paclitaxel on c-Abl kinase and other signaling pathways. In addition, we investigated the effects of c-Abl kinase on paclitaxel toxicity and on paclitaxel-induced G2/M arrest.

### MATERIALS AND METHODS

### Cell culture

*c-abl*-null mouse fibroblasts (3T3-$Abl^{-/-}$ cells), polyclonal populations of reconstituted 3T3-$Abl^{+}$ cells, and 3T3 mock infected cells (3T3-mock cells) were obtained from Dr JYJ Wang (Wang, 1993). 3T3-$Abl^{+}$ and 3T3-mock cells were obtained by infection and selection of 3T3-$Abl^{-/-}$ cells with *c-abl* or empty recombinant retroviruses containing the hygromycin-resistance gene. 3T3-mock cells were used as a negative control in all experiments. All cell lines were maintained in Dulbecco's modified Eagle's medium (Irvine Scientific, Irvine CA, USA) supplemented with 100 mM L-glutamine and 10% heat-inactivated foetal bovine serum. The absence and presence of expression of c-Abl in 3T3-$Abl^{-/-}$, 3T3-mock, and 3T3-$Abl^{+}$ fibroblasts was verified by immunoblot analysis (data not shown).

### Reagents

Paclitaxel, vinblastine, vincristine, etoposide, and doxorubicin were purchased from Sigma Chemical Co (St. Louis MO, USA) and dissolved in DMSO. Docetaxel was purchased from

*Received 21 December 1999*
*Revised 28 June 2000*
*Accepted 30 June 2000*

*Correspondence to:* RD Christen

Rhone-Poulenc Rorer Pharmaceuticals Inc (Collegeville PA, USA), and was prepared as a 50 mM stock solution in polysorbate 80. Vinorelbine was obtained from Glaxo Wellcome Inc (Research Triangle Park NC, USA), a stock solution of 9.26 mM was prepared in water. Cisplatin was obtained from Bristol-Myers Squibb Co (Princeton NJ, USA). Cisplatin was dissolved in 0.9% NaCl as 3.33 mM stock solution.

### Growth rate assay

Since 3T3 cells do not form discrete colonies, sulforhodamine B growth rate assays were performed. Fibroblasts were seeded into 96-well plates at a density of 1500 cells well$^{-1}$ in 100 µl medium. After 24 h, drugs were added in a final volume of 100 µl medium, and cells were exposed to microtubule-damaging agents for 24 h. Cells were allowed to grow for an additional 72 h after the beginning of drug exposure. Cell growth was stopped by adding 50 µl of 50% (w/v) trichloroacetic acid, and cellular proteins were stained with sulforhodamine B (Sigma Chemical Co) and measured by spectrophotometry (Skehan et al, 1990). Control plates were fixed to estimate the amount of cellular proteins at time 0. The relative growth rate, *r*, was calculated as reported previously (Monks et al, 1991). Each experiment was performed in triplicate, and $IC_{50}$ values were estimated by linear interpolation at $r = 0.5$. The growth rates of untreated Abl-proficient and -deficient cells were identical.

### Quantitation of apoptotic cells

3T3 cells were treated with 160 nM paclitaxel for 24 h. 72 h after treatment, cells were collected by trypsinization and resuspended in PBS containing 4 µg ml$^{-1}$ acridine orange and 4 µg ml$^{-1}$ ethidium bromide. Subsequently, cells were assessed for apoptotic morphology by supravital fluorescence microscopy. Cells were scored as apoptotic according to established morphologic criteria (McGahon et al, 1995).

### Cell cycle phase distribution

Approximately 1–2 × $10^6$ cells were exposed to an equimolar concentration of 160 nM paclitaxel for 24 h. This corresponded to an $IC_{40}$ and an $IC_{70}$ concentration in 3T3-mock cells and 3T3-Abl$^+$ cells, respectively. At 0, 1, 2, 3, 4, 5 and 6 days after starting paclitaxel treatment, cells were harvested by trypsinization, washed twice with ice-cold PBS, and fixed in ice-cold 70% ethanol at a concentration of $10^6$ cells ml$^{-1}$. Cells were counted and $10^6$ cells per sample were centrifuged, resuspended in 300 µl of ice-cold PBS and treated with 0.1 mg ml$^{-1}$ RNase A (Sigma Chemical Co) at 37°C for 30 min. Propidium iodide (Molecular Probes, Eugene OR, USA) at a final concentration of 50 µg ml$^{-1}$ was then added to the cell suspensions. After a 30 min incubation on ice, cells were analysed on a FACScan flow cytometer (Becton-Dickinson, San Jose CA, USA). Multicycle AV Cell Cycle software (Phoenix Flow Systems, San Diego CA, USA) was used to calculate the fraction of cells in each phase of the cell cycle, as described previously by Dean and Jett (1974).

### In vitro immune complex kinase assay for c-Abl kinase

Cells were grown to approximately 80% confluence and treated with an equimolar concentration of 160 nM paclitaxel for 24 h. At 12 and 24 h after the beginning of exposure to paclitaxel, cells were lysed in 1 ml lysis buffer (10 mM Tris-HCL (pH 7.4), 150 mM NaCl, 5 mM EDTA, 1% Triton X-100, 5 mM DTT, 1 mM sodium vanadate, 0.1 mM phenylmethylsulfonyl fluoride, and 5 mM aminocaproic acid) for 30 min on ice. The insoluble material was removed by centrifugation at 30 000 ***g*** for 20 min at 4°C. Equal amounts of protein were incubated with protein A-Sepharose and anti-Abl (2.5 µg) (K-12, Santa Cruz Biotechnology, Santa Cruz CA, USA) for 3 h at 4°C. Anti-Abl immune complexes were washed twice with lysis buffer (500 mM NaCl, lysis buffer/100 mM NaCl) and twice with kinase buffer (25 mM Tris-HCl (pH 7.4), 10 mM $MgCl_2$, and 1 mM DTT). The washed pellet was resuspended in 20 µl of kinase buffer with 50 µCi (γ-$^{32}$P) ATP (3000 Ci mmol$^{-1}$), 10 µM cold ATP and 1 µg of GST-CTD as substrate and incubated for 30 min at room temperature (Baskaran et al, 1996). Reactions were terminated by the addition of the SDS sample buffer and boiling for 5 min. The phosphorylated proteins were resolved by 5–15% SDS-PAGE and visualized by autoradiography. The amounts of c-Abl protein in the anti-Abl immunoprecipitates were analysed by immunoblotting with anti-Abl (8E9; Pharmingen, San Diego CA, USA). The antigen–antibody complexes were visualized by enhanced chemiluminescence.

### Western blotting

Cells growing in log phase were treated with 160 nM paclitaxel for 24 h. At various points in time, cells were lysed in 100 µl lysis buffer as described above. 50 µg of total protein were subjected to electrophoresis on 8%, 15%, or 10% polyacrylamide gels, for analysis of phosphorylated MAPK, p21$^{WAF1}$ and p53, respectively. Cellular proteins were transferred to a polyvinylidene difluoride membrane (Immobilon P; Millipore, Bedford MA, USA) by electroblotting. Membranes were exposed to polyclonal anti-phospho-MAPK antibody (New England Biolaboratories Inc, Beverly MA, USA), polyclonal anti-p21$^{WAF1}$ antibody (Santa Cruz Biotechnology, Santa Cruz CA, USA), or monoclonal anti-p53 antibody (Santa Cruz Biotechnology), diluted 1: 1000 in 3–5% bovine serum albumin. After washing, blots were exposed to horseradish peroxidase-conjugated anti-rabbit or anti-mouse antibodies diluted 1:3000 (Amersham Life Science Inc, Arlington Heights IL, USA) and the complexes were visualized by enhanced chemiluminescence.

### RESULTS

### Effect of paclitaxel on c-Abl kinase activity

DNA damage has been shown to activate c-Abl tyrosine kinase (Kharbanda et al, 1995*a*; 1995b; Liu et al, 1996; Nehme et al, 1997). We were interested in whether c-Abl tyrosine kinase is also involved in the cellular response to microtubule damage. 3T3-mock and 3T3-Abl$^+$ cells were treated with 160 nM paclitaxel for 24 h, and anti-Abl immunoprecipitates were prepared from cell lysates at 12, and 24 h. In vitro kinase assays were performed with the GST-CTD protein as substrate (Baskaran et al, 1996). GST-CTD phosphorylation was not detectable in untreated cells (Figure 1). In Abl-proficient cells, treatment with paclitaxel resulted in activation of c-Abl kinase at 12 and 24 h. As expected, paclitaxel did not activate c-Abl kinase in Abl-deficient cells. Paclitaxel had no

© 2000 Cancer Research Campaign 

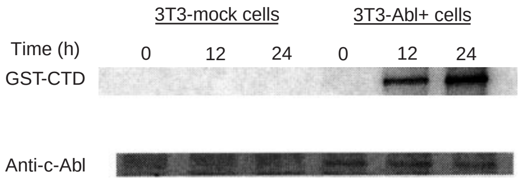

**Figure 1** Effect of paclitaxel on c-Abl kinase activity. 3T3 cells were exposed to 160 nM paclitaxel and harvested at 12 and 24 h after the beginning of drug exposure. Time point 0 h represents untreated control cells. Upper panel, activation of c-Abl determined by an immune complex kinase assay with GST-CTD as the substrate. Lower panel, Western blot analysis using an anti-Abl antibody. The experiment was repeated three times with similar results detectable effect on the levels of c-Abl protein in 3T3 cells (Figure 1, lower panel).

### Effect of c-Abl kinase on paclitaxel sensitivity

In order to understand the role of c-Abl in the cellular response to microtubule damage, we determined the effect of c-Abl kinase on the sensitivity of 3T3 cells to microtubule-damaging agents (Table 1 and Figure 2). Abl-proficient cells were 3.7 ± 0.5-fold more sensitive to paclitaxel relative to Abl-deficient cells ($IC_{50}$ 70 ± 20 vs 260 ± 50 nM, mean ± SD, $n = 4$; $P = 0.004$ by two-sided t-test). The effect of c-Abl on the sensitivity of 3T3 cells to docetaxel was much less marked and was not statistically significant. The presence of c-Abl kinase had no significant effect on the sensitivity of 3T3 cells to other microtubule-damaging agents, including vincristine, vinblastine, and vinorelbine. Similarly, c-Abl kinase had no effect on the sensitivity of 3T3 cells to cisplatin, etoposide and doxorubicin (data not shown).

### Effect of c-Abl kinase on paclitaxel-induced apoptosis

Paclitaxel has been shown to induce apoptosis in fibroblasts and tumour cells (Fan, 1999). We have confirmed that paclitaxel triggers apoptosis in 3T3 cells by demonstrating paclitaxel-induced cleavage of poly (ADP-ribose) polymerase (PARP), a substrate for caspase 3 (Nicholson et al, 1995) (data not shown). In order to determine the effect of c-Abl kinase on paclitaxel-induced apoptosis, 3T3-mock and 3T3-$Abl^+$ cells were treated with 160 nM paclitaxel for 24 h and apoptotic cells were quantitated 72 h after the beginning of drug exposure. The presence of c-Abl resulted in a 1.9 ± 0.3 (mean ± SD)-fold increase in apoptotic cells in response to paclitaxel treatment ($n = 3$, $P = 0.03$ by two-sided t-test). Interestingly, c-Abl had no effect on the number of apoptotic cells in response to treatment with 4 nM docetaxel for 24 h (data not shown).

### Effect of c-Abl kinase and paclitaxel on cell cycle phase distribution

In order to understand the mechanism(s) by which c-Abl kinase regulates the sensitivity to paclitaxel, we determined the effect of paclitaxel on cell cycle phase distribution in Abl-proficient and -deficient cells (Figure 3). Cells were exposed to 160 nM paclitaxel for 24 h which corresponded to an $IC_{40}$ and an $IC_{70}$ concentration in 3T3-mock and 3T3-$Abl^+$ cells, respectively. Paclitaxel caused a marked G2/M arrest which peaked at 24 h after the beginning of drug exposure. At 24 h, the fraction of cells in G2/M phase was 82 ± 9% and 74 ± 12% in Abl-proficient and Abl-deficient cells, respectively (mean ± SD, $n = 3$, $P < 0.005$ by two-sided t-test). In Abl-proficient cells, the G2/M arrest persisted for a considerably longer period of time relative to Abl-deficient cells. The difference in the fraction of G2/M cells between Abl-proficient and Abl-deficient cells was most marked at 6 days after starting exposure to paclitaxel (66 ± 6% vs 32 ± 5%, mean ± SD, $n = 3$, $P = 0.002$ by two-sided t-test).

### Effect of c-Abl kinase on paclitaxel-induced G2/M arrest

In order to better quantitate the effect of c-Abl kinase on paclitaxel-induced G2/M arrest, we calculated the area under the curve for fraction of cells in each phase of the cell cycle over time (percent × days). The AUC for the G2/M phase of paclitaxel treated cells was 260 ± 40 and 400 ± 30 (percent of cells in G2/M phase × days, mean ± SD) in Abl-deficient and Abl-proficient cells, respectively (Figure 4). This represents a 1.6 ± 0.2-fold difference in AUC which is statistically significant ($n = 3$, $P = 0.008$, by two-sided t-test).

### Effect of c-Abl kinase and paclitaxel on p21WAF1 expression

Paclitaxel has been reported to induce $p21^{WAF1}$ (Blagosklonny et al, 1995), a protein known to be a general inhibitor of cyclin-dependent kinases and a modulator of proliferating-cell-nuclear antigen activity (Harper et al, 1993; Hunter and Pines, 1994). Therefore, we investigated the effect of paclitaxel on the level of expression of $p21^{WAF1}$ in 3T3-mock and 3T3-$Abl^+$ cells (Figure 5). $p21^{WAF1}$ expression was determined by immunoblotting using an anti-$p21^{WAF1}$ antibody. Cells were exposed to an equimolar concentration of paclitaxel (160 nM) for 24 h. In Abl-proficient cells,

**Table 1** Effect of the c-Abl kinase on drug sensitivity. The sulforhodamine B growth-rate assay was used to generate dose–response curves. Results represent mean ± SD of at least three independent experiments performed with triplicate cultures. The dose-modifying factor represents the ratio of the $IC_{50}$ values of 3T3-mock over 3T3-$Abl^+$ cells. $IC_{50}$ values were compared by two-sided t-test

| Drug | Exposure time (h) | 3T3-mock cells ($IC_{50}$) | 3T3-$Abl^+$ cells ($IC_{50}$) | Dose-modifying factor | *P*-value |
|---|---|---|---|---|---|
| Paclitaxel (nM) | 24 | 260 ± 50 | 70 ± 20 | 3.7 ± 0.5 | 0.004 |
| Docetaxel (nM) | 24 | 4.1 ± 1.6 | 3.8 ± 1.2 | 1.1 ± 0.3 | 0.808 |
| Vinblastine (nM) | 24 | 43 ± 5 | 33 ± 8 | 1.3 ± 0.4 | 0.14 |
| Vincristine (µM) | 24 | 1.8 ± 0.4 | 1.5 ± 0.5 | 1.2 ± 0.3 | 0.46 |
| Vinorelbine (nM) | 24 | 85 ± 15 | 77 ± 21 | 1.1 ± 0.2 | 0.62 |

© 2000 Cancer Research Campaign

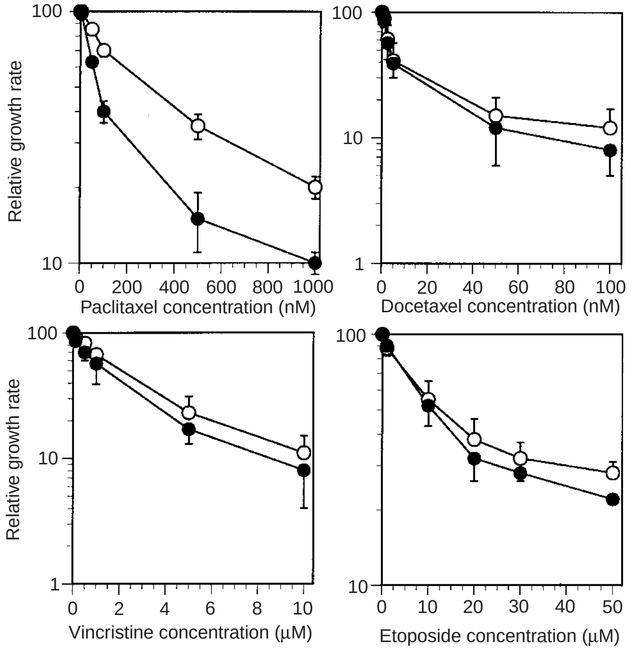

**Figure 2** Effect of c-Abl kinase on drug sensitivity. The sulforhodamine B growth-rate assay was used to determine the effect of anticancer drugs in 3T3 cells. ● = 3T3-Abl⁺ cells; ○ = 3T3-mock cells. Data points represent mean ± SD of at least three independent experiments performed with triplicate cultures

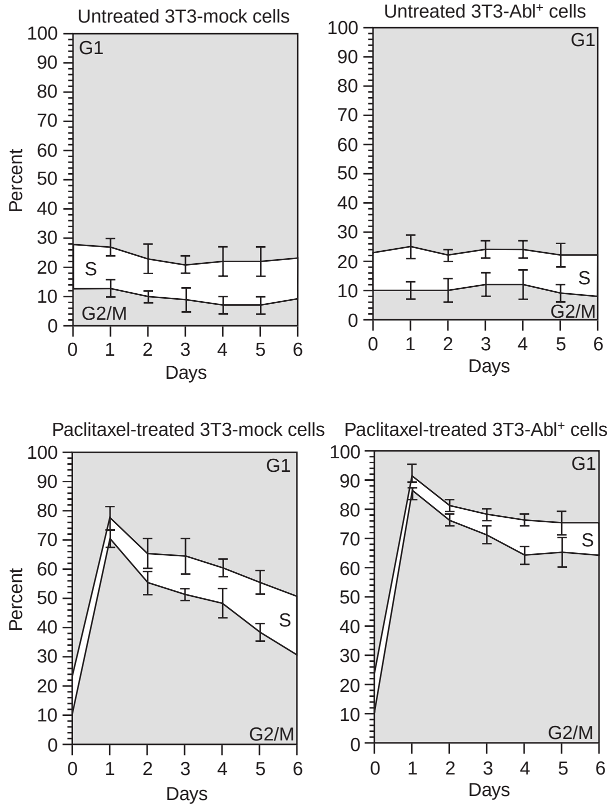

**Figure 3** Effect of c-Abl kinase and paclitaxel on cell-cycle phase distribution. Cells were exposed to 160 nM paclitaxel for 24 h and harvested at the indicated times. The cell cycle phase distribution was determined by flow cytometry. Data points represent mean ± SD of three different experiments paclitaxel caused a rapid and marked induction of p21$^{WAF1}$ which peaked at 24 h. In Abl-deficient cells, however, paclitaxel had no effect on the level of expression of p21$^{WAF1}$ at 24 h; at later points in time, overexpression of p21$^{WAF1}$ was observed.

### Effect of c-Abl kinase and paclitaxel on p53 expression p53 has been shown to regulate the expression of p21$^{WAF1}$ (El-Deiry et al, 1993). In paclitaxel-treated HCT-116 cells, p53 expression remained unchanged during the first 6 h, increased at 12 and 18 h, peaked at 24 h, and subsequently decreased rapidly (Stewart et al, 1999). To determine whether induction of p53 was required for paclitaxel-induced overexpression of p21$^{WAF1}$, we examined the effect of paclitaxel on p53 expression in 3T3-mock and 3T3-Abl$^+$ cells (Figure 5). Cells were exposed to 160 nM paclitaxel for 24 h and cell lysates prepared at 24, 48, and 72 h were examined for p53 protein levels using an anti-p53 antibody. In three separate experiments paclitaxel had no significant effect on p53 expression in 3T3-mock and 3T3-Abl$^+$ cells.

### Effect of c-Abl kinase and paclitaxel on MAPK activity

Paclitaxel has been shown to activate MAPK without altering the level of MAPK expression (Ding et al, 1996; Wang et al, 1998; Shtil et al, 1999). MAPK has been shown to be an upstream regulator of p21$^{WAF1}$ (Blagosklonny et al, 1995). We therefore investigated the effect of c-Abl kinase and paclitaxel on MAPK activity in 3T3-mock and 3T3-Abl$^+$ cells (Figure 5). MAPK activity was determined by immunoblotting using an antibody directed against

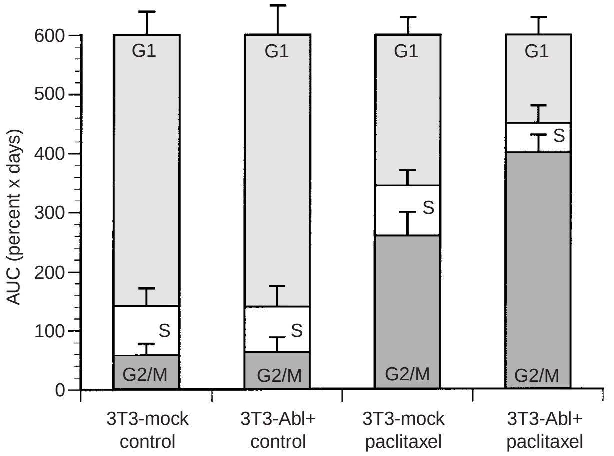

**Figure 4** Effect of c-Abl kinase on paclitaxel-induced G2/M arrest. Each phase of the cell cycle is expressed as the area under the curve of fraction of cells over time (percent × days). The AUC for the G2/M phase was 1.6 ± 0.2-fold larger in paclitaxel-treated Abl-proficient cells relative to paclitaxel-treated Abl-deficient cells ($n$ = 3, $P$ = 0.008, by two-sided $t$-test). Columns represent mean ± SD of three different experiments the phosphorylated form of MAPK. The fully phosphorylated MAPK protein served as a positive control. Cells were exposed to

© 2000 Cancer Research Campaign

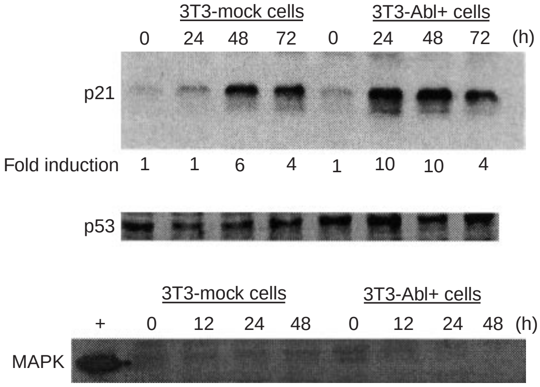

**Figure 5** Effect of c-Abl kinase and paclitaxel on p21$^{WAF1}$ and p53 expression and MAPK phosphorylation. Cells were exposed to 160 nM paclitaxel for 24 h and harvested at the indicated times after the beginning of drug exposure. Time-point 0 h represents untreated control cells. Cellular proteins were analysed by SDS PAGE and Western blotting using antibodies against p21$^{WAF1}$, p53 and the phosphorylated form of MAPK. The fully phosphorylated MAPK protein was used as a positive control (+). Experiments were repeated three times with similar results

160 nM paclitaxel for 24 h. Paclitaxel had no effect on MAPK phosphorylation in 3T3-mock and 3T3-Abl$^+$ cells. These findings indicate that, in 3T3 cells, upregulation of p21$^{WAF1}$ by paclitaxel is mediated by a pathway that is independent of MAPK.

### DISCUSSION

DNA damage caused by ionizing radiation and other DNA-damaging agents has been shown to result in activation of the c-Abl tyrosine kinase (Baskaran et al, 1997; Shafman et al, 1997). We now report that, in addition to DNA damage, microtubule damage induced by paclitaxel results in activation of c-Abl kinase. Even though both DNA damage and microtubule damage activate c-Abl kinase, the signaling pathways activated by the two types of cellular injury differ in several respects. For instance, in 3T3 cells, DNA-damaging agents activate the MAPK pathway (Nehme et al, 1997), whereas paclitaxel-induced microtubule damage did not.

Recent work has provided some insight related to the mechanism(s) by which DNA damage is detected. We have previously shown that activation of JNK and c-Abl by cisplatin is dependent on the integrity of DNA mismatch repair function, indicating that DNA mismatch repair proteins serve as a detector for DNA damage due to cisplatin (Nehme et al, 1997). The mechanism by which microtubule damage is detected, and the more upstream events that lead to activation of c-Abl in response to microtubule damage, are not understood.

In order to investigate the role of c-Abl in the cellular response to microtubule damage, we determined the effect of c-Abl kinase on the sensitivity of 3T3 cells to microtubule-damaging agents and found that loss of c-Abl kinase confers resistance to paclitaxel. Several mechanisms of acquired paclitaxel resistance have been described in cells made resistant by prolonged treatment at low drug concentrations. Paclitaxel-resistant CHO cells with structurally altered $\alpha$ and/or $\beta$ tubulin and impaired ability to polymerize tubulin dimers into microtubules have been described (Cabral and Barlow, 1991). A second mechanism of acquired paclitaxel resistance fits the general pattern of multiple drug resistance (MDR) (Horwitz et al, 1993). Our results indicate that loss of c-Abl kinase is another mechanism resulting in resistance to paclitaxel.

Interestingly, c-Abl had no effect on the sensitivity of 3T3 cells to docetaxel. This finding is intriguing, since the chemical structures of paclitaxel and docetaxel are very similar, and since both are anti-microtubule agents that promote accelerated assembly of excessively stable microtubules, thus affecting microtubule-dependent cellular functions like the control of mitosis and intracellular transport (Rowinsky and Donehower, 1996). However, the two taxanes differ in several respects. Docetaxel has a higher affinity for microtubules compared with paclitaxel (Lavelle et al, 1995). In preclinical models, paclitaxel and docetaxel differ with respect to in vitro cytotoxicity and tumour cell-kill in xenograft models (Schimming et al, 1999). Furthermore, in prostate cancer cells, docetaxel is capable of inducing bcl-2 phosphorylation and apoptotic cell death at 100-fold lower concentrations than paclitaxel (Haldar et al, 1997).

In addition to regulating the sensitivity of 3T3 cells to paclitaxel, c-Abl had marked effects on the cell cycle arrest induced by paclitaxel. In Abl-proficient cells, paclitaxel induced a marked and prolonged G2/M arrest. In Abl-deficient cells, paclitaxel induced a G2/M arrest that was much less pronounced and considerably shorter in duration. The presence of c-Abl kinase resulted in a 1.6-fold increase in paclitaxel-induced G2/M arrest, as quantitated by the ratio of the area under the curve of the fraction of cells in G2/M phase over time, indicating that the extent and duration of the paclitaxel-induced G2/M arrest are dependent on c-Abl tyrosine kinase activity.

In order to understand the mechanism by which c-Abl regulates the G2/M arrest caused by microtubule damage, we determined the effect of c-Abl and paclitaxel on p21$^{WAF1}$ expression. In Abl-proficient cells, paclitaxel caused a rapid and marked induction of p21$^{WAF1}$ which peaked at 24 h. Similar time courses of p21$^{WAF1}$ induction by paclitaxel have been described in human breast and prostate cancer cells (Blagosklonny et al, 1995). In Abl-proficient cells, the time course of paclitaxel-induced overexpression of p21$^{WAF1}$ corresponded well with the time course of paclitaxel-induced G2/M arrest which both peaked at 24 h. In Abl-deficient cells, paclitaxel caused no overexpression of p21$^{WAF1}$ at 24 h; however, at later points in time, overexpression of p21$^{WAF1}$ was observed. These findings indicate that c-Abl promotes the G2/M arrest in response to paclitaxel-induced microtubule damage by regulating the expression of p21$^{WAF1}$.

In Abl-deficient cells, paclitaxel caused a G2/M arrest at 24 h without inducing p21$^{WAF1}$ expression. This observation can be explained by the activation of pathways that are independent of c-Abl and p21$^{WAF1}$. For instance, cellular injury has been shown to induce the ATM-mediated phosphorylation of chk2 as well as phosphorylation of chk1, both of which phosphorylate cdc25C, promoting its cytoplasmic sequestration (Shapirow and Harper, 1999). As a consequence, cdc25C cannot dephosphorylate cdc2, which remains in an inactive state, resulting in G2/M arrest.

The increase in p21 level at 48 and 72 h in paclitaxel-treated, Abl-deficient cells can be explained by the activation of Abl-independent pathways. For instance, increased p21$^{WAF1}$ mRNA stability has been demonstrated in RKO cells following cellular injury (Wang et al, 2000). Investigation into the mechanisms

 *© 2000 Cancer Research Campaign*

underlying this stabilization process revealed that proteins present in cytoplasmic lysates formed complexes with p21$^{WAF1}$ mRNA that were inducible in response to cellular injury. The RNA-binding protein HuR was identified within p21 mRNA–protein complexes (Wang et al, 2000).

In 3T3 cells, induction of p21$^{WAF1}$ by paclitaxel was not associated with overexpression of p53. This finding is in line with other reports, indicating that p21$^{WAF1}$ may be induced by p53-independent pathways. For instance, p53-independent induction of p21$^{WAF1}$ was observed in other cell lines, including breast and prostate carcinoma cells treated with paclitaxel (Blagosklonny et al, 1995), human breast carcinoma cells treated with etoposide (Sheikh et al, 1994), HaCat cells exposed to transforming growth factor β (Datto et al, 1995), and in KG-1 cells treated with γ-rays or tumour-necrosis factor α (Akashi et al, 1995).

One potential mechanism by which paclitaxel regulates the expression of p21$^{WAF1}$ is the MAPK signaling pathway. Two recent studies have reported that activation of the MAPK protein is involved in induction of p21$^{WAF1}$ in response to oxidative stress (Esposito et al, 1997), and to paclitaxel (Blagosklonny et al, 1995). Raf-1 kinase, a principal upstream regulator of MAPK activity, has been shown to be responsible for paclitaxel-induced overexpression of p21$^{WAF1}$ (Blagosklonny et al, 1995). However, our results in 3T3 cells show that induction of p21$^{WAF1}$ by paclitaxel was not mediated by the MAPK pathway, since paclitaxel had no significant effect on MAPK phosphorylation.

We conclude that, in addition to DNA damage, paclitaxel-induced microtubule damage results in activation of c-Abl kinase. c-Abl kinase plays an important role in the cellular response to microtubule damage; c-Abl kinase regulates the sensitivity of 3T3 cells to paclitaxel and regulates paclitaxel-induced G2/M arrest in a pathway that is dependent on p21$^{WAF1}$.

### ACKNOWLEDGEMENTS

This work was supported by grant 4154 from the Council for Tobacco Research, and by grants from CaPCURE and the Colleen Gilbert Foundation. This work was conducted in part by the Clayton Foundation for Research–California Division. Drs Lin and Christen are Clayton Foundation Investigators. The authors wish to thank Dr JYJ Wang, Department of Biology, University of California San Diego, for generously providing Abl-proficient and -deficient 3T3 cells.

### REFERENCES

Akashi M, Hachiya M, Osawa Y, Spirin K, Suzuki G and Koeffler HP (1995) Irradiation induces WAF1 expression through a p53-independent pathway in KG-1 cells. *J Biol Chem* **270**: 19181–19187

Baskaran R, Chiang GG and Wang JYJ (1996) Identification of a binding site in c-Abl tyrosine kinase for the C-terminal repeated domain of RNA polymerase II. *Mol Cell Biol* **16**: 3361–3369

Baskaran AR, Wood LD, Whitaker LL, Canman CE, Morgan SE, Xu Y, Barlow C, Baltimore D, Wynshaw-Boris A, Kastan MB and Wang JYJ (1997) Ataxia telangiectasia mutant protein activates c-Abl tyrosine kinase in response to ionizing radiation. *Nature* **387**: 516–519

Blagosklonny MV, Schulte TW, Nguyen P, Mimnaugh EG, Trepel J and Neckers L (1995) Taxol induction of p21$^{WAF1}$ and p53 requires c-*raf*-1. *Cancer Res* **55**: 4623–4626

Cabral FR and Barlow SB (1991) Resistance to the antimitotic agents as genetic probes of microtubule structure an function. *Pharmacol Ther* **52**: 159–162

Datto MB, Li Y, Panus JF, Howe DJ, Xiong Y and Wang X-F (1995) Transforming growth factor beta induces the cyclin-dependent kinase inhibitor p21 through a p53-independent mechanism. *Proc Natl Acad Sci USA* **92**: 5545–5549

Dean PN and Jett JH (1974) Mathematical analysis of DNA distributions derived from flow microfluorometry. *J Cell Biol* **60**: 523–527

Ding A, Chen B, Fuortes M and Blum E (1996) Association of mitogen-activated protein kinases with microtubules in mouse macrophages. *J Exp Med* **183**: 1899–1904

El-Deiry WS, Tokino T, Velculesku VE, Levy DB, Parsons R, Trent JM, Lin D, Mercer WE, Kinzler KW and Vogelstein B (1993) WAF1, a potential mediator of p53 tumor suppression. *Cell* **75**: 817–825

Esposito F, Cuccovillo F, Vanoni M, Cimino F, Anderson CW, Appella E and Russo T (1997) Redox-mediated regulation of p21$^{waf1/cip1}$ expression involves a post-transcriptional mechanism and activation of the mitogen-activated pathway. *Eur J Biochem* **245**: 730–737

Fan W (1999) Possible mechanisms of paclitaxel-induced apoptosis. *Biochem. Pharmacol* **57**: 1215–1221

Goga AL, X Hambuch TM, Senechal K, Major E, Berk AJ, Witte ON, Sawyers CL (1995) p53 dependent growth suppression by the c-Abl nuclear tyrosine kinase. *Oncogene* **11**: 791–799

Haldar S, Basu A and Croce CM (1997) Bcl2 is the guardian of microtubule integrity. *Cancer Res* **57**: 229–233

Harper JW, Adami GR, Wei N, Keyomarsi K and Elledge SJ (1993) The p21 Cdk-interacting protein Cip1 is a potent inhibitor of G1 cyclin-dependent kinases. *Cell* **75**: 805–816

Horwitz S, Cohen D, Rao S, Ringel I, Shen H and Yang C (1993) Taxol: mechanisms of action and resistance. *J Natl Cancer Inst Monographs* **15**: 55–61

Hunter T and Pines J (1994) Cyclins and cancer. II: cyclin D and CDK inhibitors come of age. *Cell* **79**: 573–582

Kharbanda S, Pandey P, Ren R, Mayer B, Zon L and Kufe D (1995a) c-Abl activation regulates induction of the SEK1/stress-activated protein kinase pathway in the cellular response to 1-beta-D-arabinofuranosylcytosine. *J Biol Chem* **270**: 30278–30281

Kharbanda S, Ren R, Pandey P, Shafman TD, Feller SM, Weichselbaum RR and Kufe DW (1995b) Activation of the c-Abl tyrosine kinase in the stress response to DNA-damaging agents. *Nature* **376**: 785–788

Kharbanda S, Yuan ZM, Weichselbaum R and Kufe D (1998) Determination of the cell fate by c-Abl activation in the response to DNA damage. *Oncogene* **17**: 3309–3318

Kipreos ET and Wang JYJ (1992) Cell cycle-regulated binding of c-Abl tyrosine kinase to DNA. *Science* **256**: 382–385

Lavelle F, Bissery MC, Combeau C, Riou JF, Vrignoud P and Andre S (1995) Preclinical evaluation of docetaxel. *Semin Oncol* **22**: 3–9

Liu Z-G, Baskaran R, Lea-Chou ET, Wood LD, Chen Y, Karin M and Wang JYJ (1996) Three distinct signalling responses by murine fibroblasts to genotoxic stress. *Nature* **384**: 273–276

McGahon AJ, Martin SM, Sissonnette RP, Mahboubi A, Shi Y, Mogil RJ, Nishioka WK and Green DR (1995) The end of the cell line: methods for the study of apoptosis. In *Cell Death*, Schwarz LM and Osborne BA (eds), Vol. 46. Academic Press: San Diego

Monks A, Scudiero D, Skehan P, Shoemaker R, Paull K, Vistica D, Hose C, Langley J, Cronise P, Vaigro-Wolff A, Gray-Woodrich M, Campbell H, Mayo J and Boyd M (1991) Feasibility of a high-flux anticancer drug screen using a diverse panel of cultured human tumor cell lines. *J Natl Cancer Inst* **83**: 757–766

Nehme A, Rajasekaran B, Aebi S, Fink D, Nebel S, Cenni B, Wang JYJ, Howell SB and Christen RD (1997) Differential induction of c-Jun NH2-terminal kinase and c-Abl kinase in DNA mismatch repair-proficient and -deficient cells exposed to cisplatin. *Cancer Res* **57**: 3253–3257

Nicholson DW, Ali A, Thomberry NA, Vaillancourt JP, Ding CK, Gallant M, Gareau Y, Griffin PR, Labelle M, Lazebnik YA, Munday NA, Raju SM, Smulson ME, Yamin TT, Yu VL and Miller DK (1995) Identification and inhibition of the ICE/CED-3 protease necessary for mammalian apoptosis. *Nature (Lond.)*, **376**: 37–43

Rowinsky EK and Donehower RC (1996) Antimicrotubule agents. In *Cancer Chemotherapy and Biotherapy. Principles and Practice*, Chabner BA and Longo DL (eds) pp 263–296. Lippincott-Raven Publishers: Philadelphia

Schimming R, Mason KA, Hunter N, Weil M, Kishi K and Milas L (1999) Lack of correlation between mitotic arrest or apoptosis and antitumor effect of docetaxel. *Cancer Chemother Pharmacol* **43**: 165–172

Shafman T, Khanna KK, Kedar P, Spring K, Kozlov S, Yen T, Hobson K, Gatei M, Zhang N, Watters D, Egerton M, Shiloh Y, Kharbanda S, Kufe D and Lavin MF (1997) Interaction between ATM protein and c-Abl in response to DNA damage. *Nature* **387**: 520–523

*© 2000 Cancer Research Campaign*

Shapirow GI and Harper JW (1999) Anticancer drug targets: cell cycle and checkpoint control. *J Clin Invest* **104**: 1645–1653

Sheikh MS, Li XS, Chen JC, Shao ZM, Ordonez JV and Fontana JA (1994) Mechanisms of regulation of WAF1/Cip1 gene expression in human breast carcinoma: role of p53-dependent and independent signal transduction pathways. *Oncogene* **9**: 3407–3415

Shtil AA, Mandlekar S, Yu R, Walter RJ, Hagen K, Tan TH, Roninson IB and Kong AT (1999) Differential regulation of mitogen-activated protein kinases by microtubule-binding agents in human breast cancer cells. *Oncogene* **18**: 334–384

Skehan P, Storeng R, Scudiero D, Monks A, McMahon J, Vistica D, Warren JT, Bokesch H, Kenney S and Boyd MR (1990) New colorimetric cytotoxicity assay for anticancer-drug screening. *J Natl Cancer Inst* **82**: 1107–1112

Stewart ZA, Mays D and Pietenpol JA (1999) Defective G1-S cell cycle checkpoint function sensitizes cells to microtubule inhibitor-induced apoptosis. *Cancer Res* **59**: 3831–3837

Wang JYJ (1993) Abl tyrosine kinase in signal transduction and cell-cycle regulation. *Curr Opin Genet Dev* **3**: 35–43

Wang T-H, Wang H-S, Ichijo H, Giannakakou P, Foster JS, Fojo T and Wimalasena J (1998) Microtubule-interfering agents activate c-Jun N-terminal kinase/stress-activated protein kinase through both ras and apoptosis signal-regulating kinase pathways. *J Biol Chem* **273**: 4928–4936

Wang W, Furneaux H, Cheng H, Caldwell MC, Hutter D, Liu Y, Holbrook N and Gorospe M (2000) HuR regulates p21 mRNA stabilization by UV light. *Mol Cell Biol* **20**: 760–769

Welch PJ and Wang JYJ (1993) A C-terminal protein-binding domain in the retinoblastoma protein regulates nuclear c-Abl tyrosine kinase in the cell cycle. *Cell* **75**: 779–790

Yuan ZM, Huang Y, Whang Y, Sawyers C, Weichselbaum R, Kharbanda S and Kufe D (1996) Role for c-Abl tyrosine kinase in growth arrest response to DNA damage. *Nature* **382**: 272–274

*© 2000 Cancer Research Campaign*
